# Equivalent short forms of the Situational Feature Recognition Test 2: Psychometric properties and analysis of interform equivalence and test–retest reliability

**DOI:** 10.1002/mpr.1802

**Published:** 2019-09-30

**Authors:** Ainara Gómez‐Gastiasoro, Javier Peña, Leire Zubiaurre‐Elorza, Naroa Ibarretxe‐Bilbao, Natalia Ojeda

**Affiliations:** ^1^ Department of Methods and Experimental Psychology, Faculty of Psychology and Education University of Deusto Bilbao Spain

**Keywords:** equivalent forms, schizophrenia, short forms, social cognition, social perception

## Abstract

**Objective:**

To obtain two equivalent short forms of the “Situational Feature Recognition Test, Version 2,” a social perception test, and their psychometric properties.

**Methods:**

Patients with schizophrenia (*n* = 101) were assessed at two different times. Statistical analyses were performed as follows: (1) Cronbach's alpha was used to assess reliability; (2) Spearman correlations, Wilcoxon signed‐rank test, and a 2 (form) × 2 (time) repeated measures multivariate analysis of variance were used to analyse interform equivalence; (3) Sensitivity to change was studied by a 2 (group) × 2 (time) repeated measures multivariate analysis of variance; (4) Spearman correlations were employed to assess test–retest reliability, convergent and discriminant validity, and relationship with functionality and symptoms.

**Results:**

The short forms showed good internal consistency at both times. Significant and moderate correlation between forms was found along with no statistically significant form x time interaction. Hits and false positives of both forms were moderately correlated at both times. Group x time interaction was significant especially for hits when assessing sensitivity to change. Both forms were significantly correlated with other social cognition domains and with functionality.

**Conclusions:**

Results of this study support the use of short forms of the Situational Feature Recognition Test, Version 2 especially in clinical trials and longitudinal studies among patients with schizophrenia.

## 1 INTRODUCTION

The study of social cognition (SC) among patients with schizophrenia has garnered increasing attention in recent years. This construct refers to those processes that enable inferences to be made from other people's beliefs, thoughts, or situations (Green et al., 2008; Savla, Vella, Armstrong, Penn, & Twamley, 2013). More specifically, SC generally comprises four subdomains: (a) theory of mind (ToM), (b) social perception (SP), (c) emotion processing (EP), and (d) attributional style (AS; Pinkham, 2014). However, research into some of these domains, such as SP and EP, has received less attention than others, especially in patients with schizophrenia (Savla et al., 2013).

According to an exhaustive meta‐analysis (Savla et al., 2013), SC research usually presents methodological limitations due to the assessment tools used to measure a remarkably complex construct. This is of special importance in clinical trials and longitudinal studies (Grant, Lawrence, Preti, Wykes, & Cella, 2017; Pinkham, 2014; Pinkham, Penn, Green, & Harvey, 2016). The psychometric properties of many SP and other SC measures have not been fully investigated to date (Green et al., 2008; Pinkham et al., 2016). Moreover, many of the assessment tools have not been tested for their use as repeated measures and some of their properties, such as test–retest reliability or possible learning effects, have not been studied at all. These limitations compromise their use, particularly in clinical trials and longitudinal studies (Grant et al., 2017; Green et al., 2008). In addition, the relationship between some SC measures and patients' functionality is not usually assessed, even though it is well known that performance on SC and especially SP might provide important data for the study of the patient's functional outcomes (Fett et al., 2011).

SP refers to the ability to decode and interpret social cues in others by integrating information about the social context and knowledge in order to make a judgement about others' behaviours (Pinkham, 2014). This ability is very necessary when stimulus interpretation is ambivalent or confusing based on the stimulus itself (e.g., tears can be interpreted as signs of sadness or joy depending on the context but can be rarely interpreted correctly if they are solely based on the stimulus itself). SP is highly related to “situational schemata” a term proposed by Corrigan and Green (1993), to refer to the interpersonal information acquired from the situation *per se* that guides interpersonal responses to a specific stimulus (Corrigan & Green, 1993). As far as the authors are aware, to date, only three of the SP measures commonly used to assess patients with schizophrenia have equivalent forms: The Social Attribution Test‐Multiple Choice (SAT‐MC; Bell, Fiszdon, Greig, & Wexler, 2010), The Awareness of Social Inferences Test (McDonald, Flanagan, Rollins, & Kinch, 2003), and The Trustworthiness/Approachability Task (Adolphs, Tranel, & Damasio, 1998). Equivalent forms of a test are tests created to measure the same construct, which are as similar as possible in terms of the distribution of item difficulty and item content, with high intercorrelation between them (Kelley, 1942). Equivalent forms have an important role to play, especially in clinical trials and longitudinal studies, in order to avoid learning effects without the need to change the measure used at different times of assessment. None of the three SP assessment tools initially proposed by the Social Cognition Psychometric Evaluation (SCOPE) study for identifying and improving the existing SC measures in schizophrenia have an alternative equivalent form, which illustrates the paucity of equivalent forms among SP tests (Pinkham et al., 2016). A new SP measure with equivalent forms was later included in the SCOPE final validation study: the SAT‐MC (Bell et al., 2010). However, this instrument showed poorer psychometric properties when compared with those used for assessing the rest of SC domains (Pinkham, Harvey, & Penn, 2018), leaving SP domain without a recommended task to assess it. The lack of SP tests with reliable equivalent forms can be also noted by observing the measures included in other recent reviews and meta‐analysis studies (Grant et al., 2017; Savla et al., 2013). From the nine different SP measures included in a review of SC clinical trials on schizophrenia, no equivalent validated forms were available (Grant et al., 2017). Similarly, in the meta‐analysis mentioned above (Savla et al., 2013), that examined the deficits of all SC domains in schizophrenia, only one measure presented an equivalent form (The Trustworthiness/Approachability Task; Adolphs et al., 1998), among the more than 10 SP measures that were included.

An additional challenge for SP assessment among patients with schizophrenia is the time needed to administer the measure. In general, current SP assessment tasks may take from between 20 to 35 min to be performed, as with the cases of the Half Profile of Nonverbal Sensitivity (PONS, Ambady, Hallahan, & Rosenthal, 1995), the Interpersonal Perception Task‐15 (Costanzo & Archer, 1989), and the Relationships Across Domains task (Sergi et al., 2009). Taking into account the overall examination time that an exhaustive neuropsychological assessment usually involves, this time might be excessive for participants, resulting in their performance being compromised.

All the limitations listed above are found not only in English instruments but also in tools in other languages. As previously noted elsewhere (Gómez‐Gastiasoro, Peña, Zubiaurre‐Elorza, Ibarretxe‐Bilbao & Ojeda, 2018), most SP measures lack Spanish adaptations and validations.

**Figure 1:**
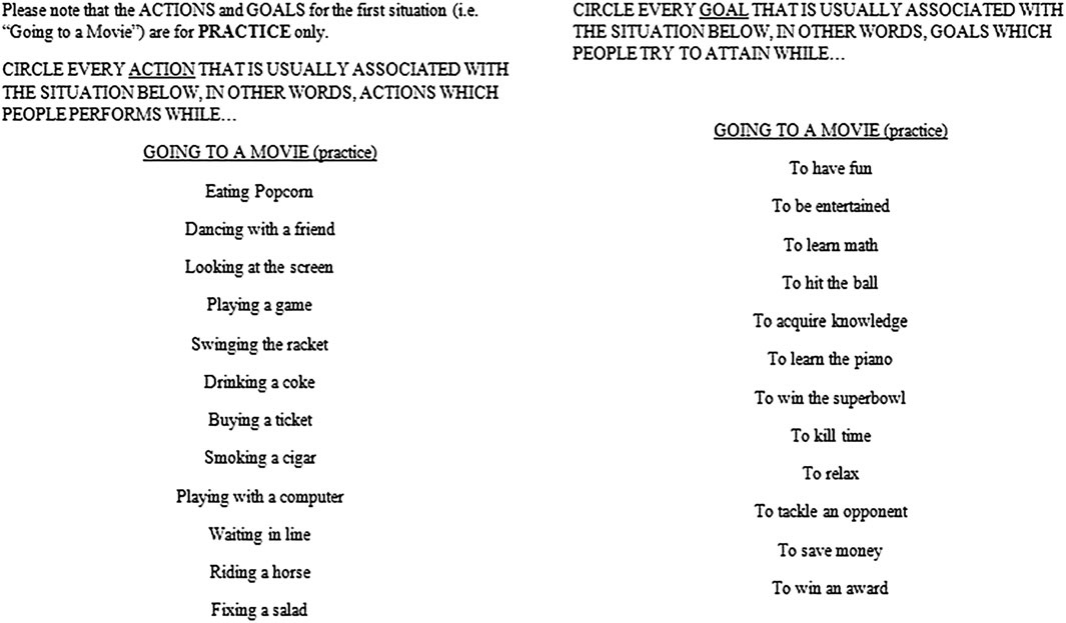
Situational Feature Recognition Test‐2 sample item (English)

**Figure 2:**
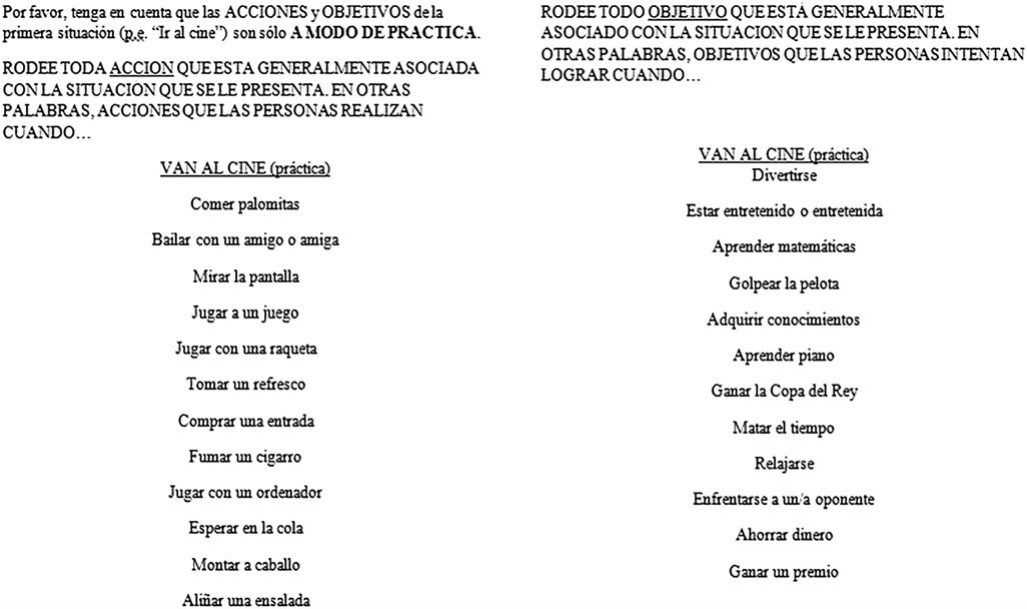
Situational Feature Recognition Test‐2 sample item (Spanish)

One of the SP tests commonly used among patients with schizophrenia that has also shown good psychometric properties is the Situational Feature Recognition Test 2 (SFRT‐2; Corrigan & Green, 1993; Corrigan, Silverman, Stephenson, Nugent‐Hirschbeck, & Buican, 1996). This assessment tool presents nine social situations along with a list of related and unrelated actions (actions that are usually performed in a given situation) and a list of related and unrelated goals (goals that people usually try to accomplish in a given situation) for each situation (Corrigan et al., 1996; Corrigan & Green, 1993; see Figures 1 and 2 for a sample item). For each situation, the participant is asked to mark all the actions and goals that they think are related to a given situation. This assessment tool has been adapted to Spanish and validated and has obtained good psychometric properties (Gómez‐Gastiasoro, Peña, Zubiaurre‐Elorza, Ibarretxe‐Bilbao & Ojeda, 2018). However, as many of the most common used SP measures, the full version of the SFRT‐2 has no equivalent forms and takes about 15–20 min to be completed, depending on the cognitive status of the participant.

The main objective of the present study was to develop two equivalent short forms of the original SFRT‐2 test in a sample of native Spanish‐speaking patients with schizophrenia. In addition, we intended to assess the psychometric properties of the short forms from a classical test theory perspective, in terms of (1) internal consistency, (2) interform equivalence, (3) sensitivity to change, (4) test–retest reliability, (5) convergent and discriminant validity in relation to other SC measures and neurocognition scores respectively, and (6) convergent validity in relation to functional impairment and symptom severity. We hypothesized that (1) short forms would have medium to high internal consistency, as was seen the Spanish validation of the test (Gómez‐Gastiasoro, Peña, Zubiaurre‐Elorza, Ibarretxe‐Bilbao & Ojeda, 2018); (2) both short forms would be equivalent in both test and retest times; (3) short forms' scores would show sensitivity to change after a cognitive rehabilitation intervention; (4) both forms would present medium to high test–retest reliability scores; (5) both forms would correlate with other SC measures and, to a lesser extent, to neurocognition measures; and (6) both forms would show a medium to high relationship with functional outcome and symptom severity scores.

## 2 METHOD

### 2.1 Participants

One hundred and one native Spanish‐speaking patients with schizophrenia (28 women and 73 men; mean age: 39.66 [9.75]; education [years]: 10.02 [3.16]; premorbid IQ: 94.43 [9.43]) were recruited from the Osakidetza Public Mental Health Services in Bizkaia and the Psychiatric Hospital of Alava (Spain). An exhaustive neuropsychological battery of tests was used for their assessment, including the SFRT‐2 (Corrigan et al., 1996; Corrigan & Green, 1993). These were conducted at two different times, 3 months apart. Neuropsychological assessment was carried out by a trained neuropsychologist in an ambulatory environment (Psychiatric Hospital of Alava, Spain). Clinical data were obtained by clinical psychiatrists and included measures such as positive and negative symptoms (assessed by the positive and negative syndrome scale; Kay, Fiszbein, & Opler, 1987), age of onset, and medication among others. The neuropsychological battery consisted of neurocognition, SC and functionality measures, and patients performed all the tests in the same session (total time = 1 hr and 30 min). All patients had been diagnosed with schizophrenia based on the American Psychiatric Association's Diagnostic and Statistical Manual of Mental Disorders, Fourth Edition, Text Revision (American Psychiatric Association, 2000). They were excluded if there was evidence of (1) alcohol or drug abuse in the previous 30 days, (2) a previous episode of loss of consciousness (3) IQ below 75, (4) substance dependence, and/or (5) a relevant neurological or medical condition. The investigation was carried out in accordance with the latest version of the Declaration of Helsinki. The study design was reviewed and approved by the Ethics Committee at the Health Department of the Basque Mental Health System in Spain and the Ethics Committee of the University of Deusto. Participants gave their informed consent before taking part in the study and after the nature of the procedures had been fully explained.

### 2.2 Procedure

Patients were involved in a project, which originally assessed the efficacy of the REHACOP cognitive rehabilitation program for psychosis (Peña et al., 2016). From the 101 patients, 47 (12 women and 35 men; mean age: 38.91 [9.63]; education [years]: 9.94 [3.07]; premorbid IQ: 94.79 [9.36]) were assigned to the control group, as described elsewhere (Peña et al., 2016). All the patients were first assessed at the beginning of the rehabilitation program and then again after 3 months of treatment. No incentives were provided to patients (either at the beginning or at the end of the clinical trial), so neither baseline nor posttreatment performance was influenced by incentives. The same full form of the SFRT‐2 (Corrigan et al., 1996) was applied at both times. The SFRT‐2 was never administered in their short forms, and performance on this measure was not a criterion for patients' inclusion in the clinical trial.

In order to develop the two equivalent short forms of the SFRT‐2, eight situations were selected from the original nine, to obtain an even number of situations. They were separated into two different forms, each of them consisting of four situations. The selection was performed taking into account the patients' degree of familiarity with the situations, in an attempt to include two familiar and two unfamiliar situations in each of the abbreviated forms. The familiarity of the situations was assessed by means of a scale in which patients had to indicate their degree of familiarity with the situation (1 = *totally familiar*; 2 = *very familiar*; 3 = *familiar*; 4 = *neutral*; 5 = *unfamiliar*; 6 = *very unfamiliar*; 7 = *totally unfamiliar*). All the situations were classified as “familiar” except “building an igloo” and “performing surgery,” which were rated as “totally unfamiliar” and “performing an ultrasound,” which was rated as “neutral.” The “playing Monopoly” situation was discarded after a preliminary reliability analysis was performed with the same sample used for this study, as it was found that the internal consistency of the short forms decreased when this situation was used. Each of the situations maintained the 12 options for both actions and goals previously presented in the Spanish adaptation and validation of the SFRT‐2 (Gómez‐Gastiasoro, Peña, Zubiaurre‐Elorza, Ibarretxe‐Bilbao & Ojeda, 2018). The situations included in the first form were “building an igloo,” “reading in a library,” “driving a car,” and “performing an ultrasound” ( $\bar{x}$ familiarity = 4.5), whereas the second form included “taking a test,” “celebrating first communion,” “having a haircut,” and “performing surgery” ( $\bar{x}$ familiarity = 4.0).

Each of the eight situations was linked to a list of actions and goals. As in the Spanish adaptation, each list of actions and goals contained five possible hits and seven possible false positives (see the example item given in Figures 1 and 2). Performance was indexed as the total scores obtained in action hits (ranging from 0 to 20), action false positives (ranging from 0 to 28), goal hits (ranging from 0 to 20), and goal false positives (ranging from 0 to 28). Administration time for each short form was estimated at being 5 min based on the assessment of 6 independent participants who completed the short forms independently. The full forms (1 and 2) are shown in Appendices 1 and 2, respectively.

### 2.3 Functionality variables

As part of the exhaustive neuropsychological battery, patients were also assessed using two functionality measures: (a) the University of California, San Diego Performance‐Based Skills Assessment‐UPSA (Patterson, Goldman, McKibbin, Hughs, & Jeste, 2001) to assess functional competence and (b) The Global Assessment of Functioning Scale (GAF; American Psychiatric Association, 1994) to assess global functioning and psychiatric symptom severity.

### 2.4 Other cognitive and SC variables

The neuropsychological battery also included a premorbid IQ measure (Word Accentuation Test; Del Ser, González‐Montalvo, Martínez‐Espinosa, Delgado‐Villapalos, & Bermejo, 1997), designed specifically for Spanish speakers. In this test, participants are asked to read aloud some uncommon words written without the accent mark, stressing the correct syllable (Del Ser et al., 1997). Raw scores were converted into estimated full scale IQ based on Gomar et al. (2011). Other neurocognitive measures were also included, such as The Hopkins Verbal Learning Test‐Revised (HVLT‐R; Benedict, Schretlen, Groninger, & Brandt, 1998), for verbal learning and memory; the Trail Making Test, Parts A and B, for processing speed and cognitive flexibility (Reitan & Wolfson, 1985); the Stroop Color‐Word Interference Test for inhibition (Van der Elst, Van Boxtel, Van Breukelen, & Jolles, 2006); and the Calibrated Ideational Fluency Assessment (Schretlen & Vannorsdall, 2010) for verbal phonetic fluency. Other SC measures were also employed, including four stories of the Strange Stories Test for Theory of Mind (Happé, 1994), the Mayer‐Salovey‐Caruso Emotional Intelligence Test for EP (Mayer, Salovey, & Caruso, 2002), and the self‐serving bias index of the Attributional Style Questionnaire for AS (Peterson et al., 1982; Sanjuan & Magallares, 2006).

### 2.5 Statistical analyses

The Kolmogorov–Smirnov test was used to assess the distribution of the variables. A number of statistical analyses were performed depending on whether the variables were normally or non‐normally distributed. Hit and false positives composite scores were obtained in order to calculate the interform equivalence, test–retest reliability, and relationship with functionality. Some statistical analyses were performed for the first and second testing times. The analyses performed at the first testing time (Time 1) were carried out on 101 patients (patients who received cognitive rehabilitation and patients in the control group). However, the analyses performed at the second testing time (Time 2) were carried out only on the 47 patients in the control group, in order to avoid the effects of cognitive rehabilitation on the scores obtained. As the SFRT‐2 was never administered in short form, all the analyses performed were post‐hoc manipulations of data collected from the full form. All the analyses were conducted using SPSS v.23 (SPSS Inc., Chicago, IL, USA).

#### 2.5.1 Reliability

The reliability of the short forms of the SFRT‐2 was examined by assessing the internal consistency of the total action hits, goal hits, action false positives, and goal false positives separately for Time 1 (*n* = 101) and Time 2 (*n* = 47), and for both forms by means of Cronbach's alpha.

#### 2.5.2 Interform equivalence

Interform equivalence between both short forms of the SFRT‐2 was assessed by analysing the relationship between both short forms' hits and false positives at Time 1 (*n* = 101) by means of Spearman correlations. As in other studies that assess cognitive alternative forms, reliability coefficients upwards of .60–.70 were stablished as being confident of clinical usefulness and robustness (Geffen, Butterworth, & Geffen, Butterworth, & Geffen, 1994). In addition, Wilcoxon signed‐rank tests were used in order to assess the differences between hits and false positives in both actions and objectives at Time 1 (*n* = 101) and Time 2 (*n* = 47) separately. In this case, interform equivalence was stablished based on the nonsignificant differences between forms and the effect sizes obtained. Finally, a repeated measures analysis (*n* = 47) was performed by means of a 2 (form) × 2 (time) repeated measures multivariate analysis of variance (MANOVA) in order to assess the interaction between form and time factors for action hits and false positives, and goal hits and false positives independently. In this case, time was included as a within‐subjects factor (with two levels: Time 1 and Time 2) and the dichotomous variable of form (Form 1 vs. Form 2) was included as an intersubject factor. SFRT‐2 scores (action hits, action false positives, goal hits, and goal false positives) were included as variables to study. Time by form interaction was studied in order to assess the differences. For this analysis, equivalence between forms was driven by the nonsignificant differences obtained in the form x time interaction and the effect sizes obtained.

#### 2.5.3 Sensitivity to change

Sensitivity to change of both short forms was assessed by means of two 2 (group) × 2 (time) repeated measures MANOVAs (one for each form) including both the experimental group receiving cognitive rehabilitation over 3 months (*n* = 51; 16 women and 35 men; mean age = 39.76 [9.54]; education [years] = 10.18 [3.33]), and the control group (*n* = 47) as an intersubjects group factor and Time 1 and Time 2 scores as a within‐subjects time factor. Changes in hits and false positives of both forms were investigated. Sensitivity to change was stablished if the group by time interaction was statistically significant.

#### 2.5.4 Test–retest reliability

Spearman correlation analyses were performed in order to assess the relationship between hits, and false positives at Time 1, and hits, and false positives at Time 2 for both short forms separately (*n* = 47). Reliability coefficients upwards of .60–.70 were stablished as being confident of clinical usefulness and robustness (Geffen et al., 1994).

#### 2.5.5 Convergent and discriminant validity

In order to assess convergent and discriminant validity, two composite scores were calculated. The first one (*α* = 0.80) included neurocognition measures such as the HVLT‐R learning and long‐term trials, completion times for the Trail Making Test Parts A and B, interference scores for the Stroop test, and words beginning with P for the Calibrated Ideational Fluency Assessment test. The second one (*α* = 81) was created by using SC measures such as all the Mayer‐Salovey‐Caruso Emotional Intelligence Test scales (except for bias, which showed no correlation with the other indices), the total scores for the Strange Stories Test for Theory of Mind, and self‐serving bias score for the Attributional Style Questionnaire. The relationship between both forms' action and goal hits and false positives and these two composite scores was assessed by means of Spearman correlation analyses.

#### 2.5.6 Relationship with functional and symptom severity variables

Spearman correlation analyses were also used in order to assess the relationship between both short forms' hits, and false positives and functionality. Correlation analyses were performed including Forms' 1 and 2 hits, and false positives and UPSA and GAF total scores at Time 1 (*n* = 101).

## 3 RESULTS

### 3.1 Clinical and SP characteristics

Clinical characteristics and data about performance on the SFRT‐2 are shown in Table 1. Data were divided by sample for Time 1 (*n* = 101) and sample for Time 2 (*n* = 47). As expected, there were more men than women in the groups in both cases.

**Table 1:** Clinical characteristics and Situational Feature Recognition Test‐2 data

	Time 1		Time 2
Variable	*n* = 101	*n* = 47	*n* = 47
	Mean (SD)/*n* (%)	Mean (SD)/*n* (%)	Mean (SD)/*n* (%)
PANSS			
Positive	17.87 (8.57)	18.06 (8.74)	13.77 (5.88)
Negative	22.70 (9.80)	23.72 (10.48)	19.84 (8.74)
General	40.45 (10.79)	40.60 (11.21)	35.49 (9.57)
SFRT‐2 (short forms)			
Total action hits F1	16.22 (3.57)	16.15 (3.48)	15.15 (4.68)
Total goal hits F1	15.97 (3.84)	16.13 (4.06)	14.94 (4.81)
Total actions FP F1	4.74 (4.05)	4.45 (3.31)	4.43 (3.44)
Total goals FP F1	4.99 (3.57)	4.94 (3.35)	4.72 (2.92)
Total action hits F2	16.84 (3.21)	17.13 (2.85)	16.45 (4.77)
Total goal hits F2	16.80 (3.05)	16.79 (3.15)	15.77 (4.86)
Total action FP F2	3.96 (3.76)	3.77 (2.97)	3.70 (3.08)
Total goal FP F2	3.33 (3.94)	2.96 (2.90)	3.09 (2.86)

Abbreviations: FP, false positives; F1, Form 1; F2, Form 2; PANSS, Positive and negative syndrome scale; SD, standard deviation; SFRT‐2, Situational Feature Recognition Test 2.

### 3.2 Reliability

For Time 1, internal consistency scores for Form 1 ranged from *α* = 0.71 (for goal false positives) to *α* = 0.77 (for action false positives), whereas scores ranged from *α* = 0.72 (for action hits) to *α* = 0.83 (for goal false positives) for Form 2. Regarding Time 2, Form 1 internal consistency scores ranged from *α* = 0.66 (for goal false positives) to *α* = 0.88 (for actions hits), whereas Form 2 scores ranged from *α* = 0.62 (for goal false positives) to *α* = 0.92 (for action hits; see Table 2).

**Table 2:** SFRT‐2 short forms' reliability

SFRT‐2 short forms		Cronbach's alpha		
		Time 1		Time 2
		*n* = 101	*n* = 47	*n* = 47
Form 1	Action hits	*α* = 0.727	*α* = 0.701	*α* = 0.882
	Action false positives	*α* = 0.773	*α* = 0.644	*α* = 0.666
	Goal hits	*α* = 0.750	*α* = 0.782	*α* = 0.869
	Goal false positives	*α* = 0.714	*α* = 0.683	*α* = 0.661
Form 2	Action hits	*α* = 0.719	*α* = 0.593	*α* = 0.915
	Action false positives	*α* = 0.804	*α* = 0.672	*α* = 0.746
	Goal hits	*α* = 0.725	*α* = 0.756	*α* = 0.916
	Goal false positives	*α* = 0.828	*α* = 0.668	*α* = 0.620

*Note.* Cronbach's alpha coefficients for forms 1 and 2 at Times 1 and 2. Form 1 includes: “building an igloo,” “reading in a library,” “driving a car,” and “performing an ultrasound.” Form 2 includes: “taking a test” “celebrating the first communion” “getting a haircut,” and “performing surgery.” Abbreviation: SFRT‐2, Situational Feature Recognition Test 2.

### 3.3 Interform equivalence

Spearman correlations showed intercorrelations between hits and false positives of Forms 1 and 2 of the SFRT‐2 for Time 1 (hits: *ρ* = 0.76, *p* < 0.001; false positives: *ρ* = 0.78, *p* < 0.001). Wilcoxon signed‐rank tests showed no statistically significant differences between action hits of Form 1 and Form 2 at Time 1. However, statistically significant differences were found between goal hits, and action and goal false positives in Forms 1 and 2 at Time 1 and Time 2, and also in action hits of Form 1 and 2 at Time 2. Effect sizes ranged from small to large (Table 3). The 2 × 2 repeated measures MANOVA showed no significant effects for form by time interaction (action hits: *p* = 0.839; action false positives: *p* = 0.923; goal hits: *p* = 0.737; goal false positives: *p* = 0.473; see Table 4).

**Table 3:** Interform equivalence

	Form 1		Form 2		*Z*	*p*	Cohen's *d*
Variable	Mean (SD)	Median	Mean (SD)	Median			
Time 1 (*n* = 101)							
Actions hits	16.22 (3.57)	17.00	16.84 (3.21)	18.00	−1.85	.06	.26
Actions FP	4.74 (4.05)	4.00	3.96 (3.76)	3.00	−3.68	<.001	.54
Goals hits	15.97 (3.84)	17.00	16.80 (3.05)	18.00	−2.80	.01	.40
Goals FP	4.99 (3.57)	4.00	3.33 (3.94)	2.00	−6.01	<.001	.93
Time 2 (*n* = 47)							
Actions hits	15.15 (4.68)	17.00	16.45 (4.77)	18.00	−3.29	<.001	.72
Actions FP	4.43 (3.44)	4.00	3.70 (3.08)	3.00	−2.29	.02	.49
Goals hits	14.94 (4.81)	18.00	15.77 (4.86)	18.00	−2.80	.01	.60
Goals FP	4.72 (2.92)	4.00	3.09 (2.86)	2.00	−4.43	<.001	1.03

*Note.* Wilcoxon signed‐rank test analyses for differences between Form 1 and Form 2 at Time 1 and Time 2. Abbreviations: SD, standard deviation; FP, false positives.

**Table 4:** Repeated measures MANOVA analyzing interaction form x time

Variable	Form 1		Form 2				
	Time 1	Time 2	Time 1	Time 2	*F*	*p*	$\eta_{p}^{2}$
	Mean (SE)	Mean (SE)	Mean (SE)	Mean (SE)			
Action hits	16.15 (0.60)	15.15 (0.60)	17.13 (0.56)	16.02 (0.56)	0.04	0.839	0.00
Action FP	4.45 (0.49)	4.43 (0.49)	3.77 (0.44)	3.70 (0.44)	0.01	0.923	0.00
Goal Hits	16.13 (0.65)	14.94 (0.65)	16.79 (0.60)	15.77 (0.60)	0.11	0.737	0.00
Goal FP	4.94 (0.46)	4.72 (0.46)	2.96 (0.42)	3.09 (0.42)	0.52	0.473	0.01

Abbreviations: FP, false positives; MANOVA, multivariate analysis of variance; SE, standard error; $\;\eta_{p}^{2}$, partial eta squared.

### 3.4 Sensitivity to change

For Form 1, the 2 × 2 repeated measures MANOVA showed significant effects for the group by time interaction for goal hits (*p* = 0.030) and a trend to significance for action hits (*p* = 0.053). No significant interaction was shown for false positives, either in actions or in goals. Similar results were found for Form 2, with significant group x time interaction for action hits (*p* = 0.004) and a trend to significance in goal hits (*p* = 0.083). Again, no significant interaction was found for false positives in either of the lists (actions and goals; Table 5).

**Table 5:** Repeated measures MANOVA analyzing interaction group x time

	Form 1						
	Experimental group (*n* = 51)		Control group (*n* = 47)				
	Time 1	Time 2	Time 1	Time 2	*F*	*p*	$\eta_{p}^{2}$
Variable	Mean (SE)	Mean (SE)	Mean (SE)	Mean (SE)			
Action hits	16.49 (0.49)	16.78 (0.51)	16.15 (0.52)	15.15 (0.53)	3.84	0.053	0.04
Action FP	4.22 (0.44)	3.47 (0.43)	4.45 (0.46)	4.43 (0.45)	1.58	0.212	0.02
Goal hits	15.86 (0.54)	16.14 (0.56)	16.13 (0.56)	14.94 (0.59)	4.85	0.030	0.05
Goal FP	4.35 (0.40)	4.04 (0.41)	4.94 (0.42)	4.72 (0.43)	0.03	0.862	0.00

	Form 2						
	Experimental group (*n* = 51)		Control group (n = 47)				
	Time 1	Time 2	Time 1	Time 2	F	*p*	$\eta_{p}^{2}$
Variable	Mean (SE)	Mean (SE)	Mean (SE)	Mean (SE)			
Action hits	16.82 (0.41)	17.51 (0.52)	17.13 (0.42)	16.02 (0.54)	8.93	0.004	0.09
Action FP	3.45 (0.41)	3.53 (0.43)	3.77 (0.43)	3.70 (0.45)	0.08	0.785	0.00
Goal hits	17.06 (0.40)	17.06 (0.55)	16.79 (0.42)	15.77 (0.57)	3.07	0.083	0.03
Goal FP	2.88 (0.42)	2.67 (0.39)	2.96 (0.44)	3.09 (0.41)	0.35	0.557	0.00

Abbreviations: FP, false positives; MANOVA, multivariate analysis of variance; SE, standard error. $\;\eta_{p}^{2}$, partial eta squared.

### 3.5 Test–retest reliability

Spearman correlations showed significant intercorrelations between hits and false positives of Form 1 at Time 1 and Form 1 at Time 2 and also between Form 2 at Time 1 and Form 2 at Time 2. Correlations coefficients ranged from .62 to .73 (see Table 6).

**Table 6:** Test–retest reliability

	Test–retest reliability (Spearman *ρ*)
Variable	*n* = 47
Form 1‐ Hits (Time 1‐ Time 2)	0.644**
Form 1‐ FP (Time 1‐ Time 2)	0.727**
Form 2‐ Hits (Time 1‐ Time 2)	0.621**
Form 2‐ FP (Time 1‐ Time 2)	0.652**

*Note.* Spearman correlation analyses between hits and false positives composite scores of Form 1 at Time 1 and Time 2 and Form 2 at Time 1 and Time 2. Abbreviations: FP, false positives; SZ, schizophrenia.

** *P* < 0.01

### 3.6 Convergent and discriminant validity

Both forms' indices showed a significant correlation with the SC composite score with the exception of goal false positives. Correlation indices were low to medium ranging from .20 to .45. SFRT‐2 short forms were also correlated with the neurocognition composite score, but to a lesser extent. In this case, neither action nor goal false positives showed a significant correlation with the composite scores. Correlation indices were low for all the measures, ranging from .15 to .37 (see Table 7).

**Table 7:** Convergent and discriminant validity

Variable	Social cognition CS	Neurocognition CS
Form 1 actions hits	.229*	.306**
Form 1 actions FP	−.438**	−.225*
Form 1 goals hits	.277**	.292**
Form 1 goals FP	−.193	−.170
Form 2 actions hits	.223*	.281**
Form 2 actions FP	−.344**	−.149
Form 2 goals hits	.343**	.365**
Form 2 goals FP	−.449**	−.298**

*Note.* Situational Feature Recognition Test‐2 short forms' correlate analysis with social cognition and neurocognition measures. Abbreviations: CS, composite score; FP, false positives.

* *p* < 0.05.

** *p* < 0.01.

### 3.7 Relationship between short forms and functionality and symptom severity variables

Results showed that both hits and false positives of Forms 1 and 2 correlated with the UPSA total score, with coefficients ranging from 0.33 to 0.41, whereas GAF scores only correlated with the false positives for Form 1 (see Table 8).

**Table 8:** Situational Feature Recognition Test‐2 short forms' correlate analysis with functional measures and symptom severity

Variable	UPSA	GAF
Form 1 Hits	0.344**	0.125
Form 1 FP	−0.410**	−0.253*
Form 2 Hits	0.371**	0.028
Form 2 FP	−0.328**	−0.161

Abbreviations: FP, false positives; GAF, The Global Assessment of Functioning Scale; UPSA, UCSD Performance‐Based Skills Assessment.

* *p* < 0.05.

** *p* < 0.01.

## 4 DISCUSSION

This study presents two equivalent short forms of the SFRT‐2 for SP assessment in patients with schizophrenia. The two short forms showed good internal consistency both at Time 1 and Time 2. Both forms' indices were related to each other, and no differences were found between forms when considering time effects, whereas patients performed better on Form 2 than on Form 1 when time was not considered, questioning interform equivalence. Both forms showed good test–retest reliability and sensitivity to change, especially for hits scores. In addition, hits and false positives for both short forms of the SFRT‐2 proved to be related to functional outcome and other SC measures.

Internal consistency indices ranged from acceptable to excellent, similarly to those obtained in the SC measures selected by the SCOPE study (Pinkham et al., 2016), such as the Bell Lysaker Emotion Recognition Task (Bryson, Bell, & Lysaker, 1997), the Penn Emotion Recognition Text (ER‐40; Kohler et al., 2003), the Reading the Mind in the Eyes Test (Eyes; Baron‐Cohen, Wheelwright, Hill, Raste, & Plumb, 2001), the Hinting task (Corcoran, Mercer, & Frith, 1995), the Relationships Across Domains test (Sergi et al., 2009), the Trustworthiness Task (Trust; Adolphs et al., 1998), and the Awareness of Social Inferences Test (McDonald et al., 2003). These indices were also in line with those presented in the original version of the test (from *α* = .75 to *α* = .84; Corrigan et al., 1996). These internal consistency indices suggest that both SFRT‐2 short forms are reliable SP measures to assess patients with schizophrenia.

The need for standardized and validated equivalent forms of social cognitive measures, especially SP measures, has been highlighted by specialists in studies such as the SCOPE (Pinkham et al., 2018, 2016). Specifically, the present short forms of the SFRT‐2 showed interform equivalence in terms of interrelationship reliability coefficients between forms and also equivalence and stability when considering assessment time effects on form equivalence. In addition, the reliability indices obtained when correlating both forms showed to be high enough to be confident of clinical usefulness and robustness according to the equivalence assessment of other cognition measures' alternative forms (Geffen et al., 1994). In contrast, when considering performance on both forms independently of time, alternative forms showed to be nonequivalent for most of the indices as patients showed a better performance on Form 2. Magnitude of the effect sizes was low to medium for actions hits and false positives and goals hits at Time 1 and for actions false positives at Time 2 but medium to high for goals false positives at Time 1 and actions and goals hits and goals false positives at Time 2, compromising interform equivalence. However, performance mean scores did not differ in more than one score from one form to another, which might be interpreted as nonclinically significant. Nevertheless, these results might point to nonequivalence between forms when assessing performance differences in each of the forms. It is difficult to compare these results to those obtained by other SC measures since, to our knowledge, only three SP assessment tools currently present alternative forms. One of those measures, the SAT‐MC, also showed differences between forms when patients' performance in both alternative forms was compared in the last SCOPE study (Pinkham et al., 2018) but not in the original manuscript which presented the alternative forms (Johannesen, Fiszdon, Weinstein, Ciosek, & Bell, 2018). However, correlation between SAT‐MC forms was not assessed in the SCOPE study (Pinkham et al., 2018), although it was studied in the original manuscript, with good outcomes (Johannesen et al., 2018). The apparent variability between the three different analyses used to assess interform equivalence is also hard to compare with other studies, since the three analyses are very rarely reported jointly for the same assessment tool. However, similar interform equivalence has been obtained by other test forms assessing verbal memory, such as the HVLT‐R (Benedict et al., 1998), a well‐recognized neurocognition measure that has been recommended for neuropsychological assessment in clinical trials of patients with schizophrenia by the MATRICS initiative (Nuechterlein et al., 2008).

In addition, hit scores of both forms showed changes after the intervention, but results were far from significant for false positives. The lack of significance for false positive responsiveness could indicate that these indices presented some kind of ceiling effect, preventing them from being sensitive to changes after a cognitive intervention. However, changes did not follow the expected pattern when assessing sensitivity to change (stability in the control group and improved scores at Time 2 in the experimental group). In this case, changes were given due to stability on the SFRT‐2 scores in the experimental group and a decrease of these scores in the control group. Therefore, this would not be reflecting sensitivity to change as it is commonly understood. Nevertheless, as far as authors are aware, there is lack of data about the pattern of longitudinal changes on the SFRT‐2 in patients with schizophrenia when no intervention is implemented. Therefore, it is not clear if the change observed in the control group is the typical pattern or not. As far as the utility of short forms as repeated measures is concerned, test–retest reliability indices (rho indices ranging from .62 to .73) were also similar to those obtained by the SCOPE study (Pinkham et al., 2018, 2016), SC measures (*r* indices ranging from .52 to .81). According to the SCOPE study, SC measures with test–retest reliability scores ≥ .60 are considered acceptable. This study calculated the test–retest reliability based on a longer time period (3 months) compared with the 2‐ to 4‐week test period in the SCOPE study, which could explain the minor differences in terms of test–retest reliability indices between the SCOPE study and this study. Given the lack of information about the utility of SP tests as repeated measures, as pointed out by a recent review (Grant et al., 2017), the test–retest reliability indices obtained highlight even further the utility of the present equivalent short forms of the SFRT‐2. Nevertheless, whereas similar to those obtained for measures assessed by the SCOPE study, the test–retest reliability scores were medium. Given that half of the sample was included in an intervention, these analyses were performed using only half of the whole sample, with possibly diminishing statistical power. Future studies should test this test–retest validity by including larger samples. In addition, given that the whole form of the test was administered to the entire sample and then divided into Form 1 and Form 2, the order in which the situations were administered was the same for all patients. This could have led to learning effects that may have promoted better performance in the latter situations. Future studies should assess test–retest validity of these two short forms by addressing the importance of the order in which they are administered.

Regarding convergent and discriminant validity, scores obtained by means of both of the SFRT‐2 short forms showed a low to medium relationship with other SC measures of EP, ToM and AS. These indices for convergent validity were in line with those provided by different studies about the relationship between the four different SC domains. Whereas ToM and EP seemed to be highly related to SP (Grant et al., 2017), AS has shown to have a weaker relationship with this domain (Bell et al., 2010; Mancuso, Horan, Kern, & Green, 2011). This discrepancy could have led to low to medium relationship levels between SP scores and the other SC measures' composite scores. Regarding discriminant validity, the short forms' scores showed a significant relationship with the neurocognition composite score. However, the effect sizes of this relationship were all low and, in general, lower than those found regarding the SC composite scores. These results are supported by the studies that have acknowledged the relationship between SC measures and neurocognition but describe lower relationship levels compared with the interrelation among the SC domains themselves (Mancuso et al., 2011; Sergi et al., 2007).

In addition, scores obtained in the short forms of the SFRT‐2 were shown to be related to functional and symptom severity measures, especially with functional competence scores measured by the UPSA test. The idea that SC would to some extent be related to, or even explain, some variance in functional outcome, has been well demonstrated (for a review, see Fett et al., 2011). In fact, relationship to functional outcome is one of the most important characteristics to be taken into account when choosing SC measures to be used in clinical trials (Pinkham, 2014; Pinkham et al., 2016). Regarding the short forms of the SFRT‐2, hits and false positives were found to be related to functional outcome. Relationship coefficients were moderate and similar to those obtained when assessing the relationship between SC measures selected by the SCOPE study and UPSA total scores (Pinkham et al., 2018, 2016). These results suggest that the short forms of the SFRT‐2 might be useful when trying to predict patients' functional outcome and symptom severity.

It is also noteworthy that, by using either of the two short forms of the SFRT‐2, the test administration time was reduced from 15 min (in the original version) to 5 min. The SCOPE study stated that, SC instruments presenting administration times under 10 min are described as being practical and tolerable for participants. Unlike most of the existing SP measures, the short forms of the SFRT‐2 provided reliable SP scores in 5 min. Despite the wide variety of SC tests available, administration time is still a challenge for SC assessment. As described by the SCOPE study, in some cases some SC measures administration times range from 20 to even 35 min, depending on the task (Pinkham et al., 2016). This can reduce the usefulness of the task, as well as making the assessment more unpleasant for the patient. Taking this into account, the short forms of the SFRT‐2 might represent one of the most practical available measures of SP, which could be especially useful in clinical trials or when employing a large neuropsychological battery.

Despite the good psychometric characteristics of the short forms of the SFRT‐2, some limitations of the present study merit further discussion. First, although the sample was large for analyses, performed at Time 1, sample size was reduced by half for all tests carried out at Time 2, due to the involvement of some of the patients in a rehabilitation program. Therefore, it would be appropriate to repeat the test–retest analyses with larger samples in order to replicate the present results. Second, differences obtained when comparing Forms 1 and 2 performance in Time 1 and Time 2 separately as well as magnitude of the obtained effect sizes suggest caution when employing SFRT‐2 Form 1 and Form 2 as equivalent forms. Third, given that the SFRT‐2 was assessed as a whole instead of separately for each short form, participant performance might differ when only the four situations included in each form are evaluated. Finally, the psychometric properties of the SFRT‐2 short forms and their utility as repeated measures should be assessed in other pathologies and with shorter and longer periods between assessments.

In conclusion, the short forms of the SFRT‐2 seem to be reliable and practical SP measures for assessing this SC domain in patients with schizophrenia. Their psychometric properties, and especially the good test–retest data obtained and the sensitivity to change shown by some of its indices, suggest that they are suitable to be included in clinical trials in order to assess SP performance and changes over time. This would contribute to gaining a better understanding of the effectiveness of cognitive interventions and longitudinal studies regarding SP performance in patients with schizophrenia.

## CONFLICT OF INTEREST STATEMENT

The authors report no conflicts of interest.

## AUTHORSHIP

Ainara Gómez‐Gastiasoro, Javier Peña, and Leire Zubiaurre‐Elorza have made substantial contributions to the conception and design, acquisition of data, and analysis and interpretation of data. Ainara Gómez‐Gastiasoro, Javier Peña, Leire Zubiaurre‐Elorza, Naroa Ibarretxe‐Bilbao, and Natalia Ojeda have been involved in drafting the manuscript and revising it critically for substantial intellectual content. All authors have given final approval of the version to be published.
